# Validation of an HIV whole genome sequencing method for HIV drug resistance testing in an Australian clinical microbiology laboratory

**DOI:** 10.1002/jmv.29273

**Published:** 2023-12-01

**Authors:** Frances Jenkins, Thomas Le, Rima Farhat, Angie Pinto, Azim Anzari, David Bonsall, Tanya Golubchik, Rory Bowden, Frederick J. Lee, Sebastiaan J. van Hal

**Affiliations:** 1 Department of Infectious Diseases and Microbiology, Royal Prince Alfred Hospital, Sydney, Australia; 2 The Kirby Institute, UNSW Australia, Sydney, Australia; 3 Wellcome Centre for Human Genetics, University of Oxford, Oxford, UK; 4 Department of Medical Sciences, Faculty of Medicine and Health, University of Sydney, Sydney, Australia; 5 The Walter and Eliza Hall Institute of Medical Research, Advanced Genomics Facility, Melbourne, Australia; 6 Department of Clinical Immunology and Allergy, Royal Prince Alfred Hospital, Sydney, Australia; 7 Sydney Medical School, University of Sydney, Sydney, Australia

**Keywords:** antiretroviral therapy, HIV, HIV drug resistance, HIV genotyping, NGS, whole genome sequencing

## Abstract

Detection of HIV drug resistance (HIVDR) is vital to successful anti-retroviral therapy (ART). HIVDR testing to determine drug-resistance mutations is routinely performed in Australia to guide ART choice in newly diagnosed people living with HIV or in cases of treatment failure. In 2022, our clinical microbiology laboratory sought to validate a next-generation sequencing (NGS)-based HIVDR assay to replace the previous Sanger-sequencing (SS)-based ViroSeq. NGS solutions for HIVDR offer higher throughput, lower costs and higher sensitivity for variant detection. We sought to validate the previously described low-cost probe-based NGS method (veSEQ-HIV) for whole-genome recovery and HIVDR-testing in a diagnostic setting. veSEQ-HIV displayed 100% and 98% accuracy in major and minor mutation detection, respectively, and 100% accuracy of subtyping (provided > 1000 mapped reads were obtained). Pairwise comparison exhibited low inter-and intrarun variability across the whole-genome (Jaccard index [*J*] = 0.993; J = 0.972) and the *Pol* gene (*J* = 0.999; *J* = 0.999), respectively. veSEQ-HIV met all our pre-set criteria based on WHO recommendations and successfully replaced ViroSeq in our laboratory. Scaling-down veSEQ-HIV to a limited batch size and sequencing on Illumina iSeq. 100, allowed easy implementation of the assay into the workflow of a small sequencing laboratory with minimal staff and equipment and the ability to meet clinically relevant test turn-around times. As HIVDR-testing moves from SS- to NGS-based methods and new ART drugs come to market (particularly those with targets outside the *Pol* region), whole-genome recovery using veSEQ-HIV provides a robust, cost-effective and “future-proof” NGS method for HIVDR-testing.

## 1 Introduction

HIV is a major public health concern worldwide. While Australia has a low prevalence of HIV in the general population (0.1% on 2017 estimate), HIV detection and management still play an important role in transmission prevention, especially in at-risk populations.^1,2^ HIV drug-resistance (HIVDR) testing to determine drug-resistance mutations (DRMs) is routinely performed in Australia to improve chances of treatment success by informing anti-retroviral therapy (ART) choice in newly-diagnosed people living with HIV/AIDS (PLWHA) or in cases of treatment failure.

Royal Prince Alfred Hospital in Sydney, Australia, routinely performs HIVDR testing and, from 2016 to 2021, utilized Sanger-sequencing (SS)-based commercial assay, ViroSeq HIV-1 genotyping system (Celera Diagnostics). ViroSeq includes RNA extraction and reverse-transcription polymerase chain reaction (RT-PCR) steps to generate a 1.8 kb amplicon covering parts of the polymerase (*Pol*) region including protease (PR) and partial reverse-transcriptase (RT) genes. The generated amplicons act as the SS-template to generate an approximately 1.2 kb electropherogram, which after manual curation acts as sequence input to the HIVDR database (HIVdb) available on Stanford University website (https://hivdb.stanford.edu/hivdb/by-patterns/). HIVdb identifies known DRMs and inferred levels of drug resistance to commonly used ART drugs.^3–5^ The integrase (IN) region is not covered by ViroSeq thus, when requested by the clinician, testing was referred to an external laboratory performing an SS-based “in-house” assay that utilizes HIVdb for analysis.

In 2020, our laboratory was advised ViroSeq would cease manufacture in mid-2021. We sought to find a suitable alternative with the ability to detect DRMs in samples with plasma viral load (VL) of around 2000 copies per mL (cp/mL), at similar costs and overcome several limitations of ViroSeq: (1) able to obtain results across all HIV-1 subtypes; (2) includes IN region; (3) able to detect sub-consensus variants as several studies have demonstrated that these subpopulations contribute to negative clinical outcomes^6,7^; and (4) was “future-proof” as novel ART agents become available.

Next-generation sequencing (NGS) solutions for HIVDR have been proposed as superior to SS-based methods due to higher throughput, lower costs, and improved sensitivity to detect sub-consensus variants.^8,9^ Several NGS-based HIVDR methods have been reported, however, the challenge remains finding protocols that work consistently without sampling bias across all HIV-1 subtypes and VLs.

A robust low-cost probe-based high-throughput NGS method for whole-genome HIV-1 recovery, HIVDR, and VL determination (veSEQ-HIV) was previously described.^10^ Previous evaluation of veSEQ-HIV performance in comparison to ViroSeq, showed a high degree of concordance in DRMs detected, higher sensitivity in the detection of subconsensus variants and ability to detect complete sequences in a majority of samples (70%) increasing with VL.^11^

veSEQ-HIV best met our requirements, however, was designed to run in batches of 96 samples.^10^ In our diagnostic setting, with need to meet clinically relevant turn-around times (TATs), we reduced this batch size to eight patient samples and performed sequencing on Illumina iSeq. 100. These adjustments allowed us to implement veSEQ-HIV into the workflow of our small sequencing laboratory with minimal staff and equipment and to achieve a mean TAT of 14 days (range 4–28 days) for subsequent clinical samples.

Here, we describe the validation of veSEQ-HIV in its utility for HIVDR testing as an “in-house” IVD in an Australian clinical microbiology laboratory.

## 2 Materials and Methods

### 2.1 Study population/sample selection

Sixty-six stored plasma samples were included with known DRMs from both treatment naïve and -experienced PLWHA that had undergone testing on ViroSeq (Celera Diagnostics) as per manufacturer’s instructions between 2016 and 2021. Associated VLs were obtained from laboratory records and ranged from 950 to >10^6^ cp/mL. Subtypes included B (*n* = 48), C (*n* = 3), CRF01_AE (*n* = 13), and CRF02_AG (*n* = 2). Sample characteristics are summarized in Table 1.

As ViroSeq does not cover the IN region, we included five samples that had SS-based IN testing performed at an external accredited laboratory and had Stanford HIVdb reports available. We did not have access to nucleotide sequences for these samples and only included them in our comparisons of subtype and mutation detection.

For reference, a dilution series (10^5^–25 cp/mL) of the highest AcroMetrix HIV-1 Panel standard was prepared (Thermo-Fisher Scientific). A negative control (PCR-confirmed HIV-negative plasma) was taken through the entire process (extraction to sequencing) for each run. A positive control was not included as sequencing was only performed on PCR (VL) confirmed HIV-positive samples.

### 2.2 Extraction

Methods were based on those previously described for veSEQ-HIV with minor modifications.^10^ Briefly, automated total RNA extraction was performed on NucliSENS e-MAG (Biomerieux) using the manufacturer’s general extraction protocol (input 500 µL; elution 25 µL).

### 2.3 cDNA generation and library preparation

RNA was concentrated using RNAClean XP beads (Beckman-Coulter) before quarter reactions for cDNA generation with maximal RNA input and without fragmentation were prepared using SMARTer Stranded Total RNA-Seq Kit v2 – Pico Input Mammalian Kit (Takara Biosciences).

Indexed libraries were prepared using the same kit and pooled equally by volume (eight patient and one negative control) before clean-up using 0.68x AMPure XP beads (Beckman-Coulter). DNA was quantified using Qubit HS-DNA kit and fluorometer (Thermo-Fisher Scientific), and fragment size determined using HS-D1000 on TapeStation 4200 (Agilent Technologies).

### 2.4 Capture-based hybridization, postcapture PCR, and clean-up

The pooled library was taken through capture using xGen hybridization and wash kit (Integrated DNA Technologies [IDT]) as per the manufacturer’s instructions. We used IDT to manufacture a custom probe panel containing 646 custom 120-mer biotinylated probes targeting the HIV genome across multiple subtypes. The custom probe sequences are published here for the first time with permission (Supporting Information: File S2). Postcapture PCR was performed as per the manufacturer’s instructions.

Clean-up was performed with 1.5x AMPure XP beads (Beckman Coulter). DNA was quantified using Qubit HS-DNA kit and fluorometer (Thermo-Fisher Scientific), and fragment size determined using HS-D1000 on TapeStation 4200 (Agilent Technologies). An additional post-capture PCR (six cycles) was added, and clean-up was repeated if minimum library molarity (1 nM for iSeq. 100) was not met at the final library stage.

### 2.5 Sequencing

Final libraries were prepared as per Illumina iSeq. 100 Sequencing System Product Documentation #200015511v00 (Illumina). A final loading concentration of 75pM with 2% PhiX control spike was used. Libraries were run on Illumina iSeq. 100 using iSeq. 100 i1 Reagent v2 (300-cycles) with read lengths of 2 × 150 bp.

### 2.6 Bioinformatics

Raw short-read data was processed using an “in-house” automated pipeline utilizing open-source software as described previously with minor modifications.^10^ Briefly, Kraken was used to filter out human reads, followed by adapter trimming using fastp.^12–16^ Host-depleted, filtered reads were taken forward, including generating assemblies using SPAdes which formed the inputs to assemble contigs suitable as input for SHIVER (v3).^17,18^

An updated Python 3.0 compliant SHIVER was used for analysis, setting a previously validated minimum depth of 15 against the reference genome (HBX2-HIV).^18,19^ The resulting output was run through Stanford University HIVdb to identify DRMs using sierrapy.^3–5^ Subsequent reports were generated using an “in-house” script based on the HIVdb json file output.

### 2.7 Assessment of assay performance

Validation criteria were pre-defined and based on WHO “Recommended methods for validating an in-house genotyping assay for surveillance of HIV drug-resistance” with minor modifications.^20^ In brief, criteria included 100% of known mutations must be detected and ≥90% of pairwise comparisons must be ≥98% identical. Recommended sensitivity for amplification was ≥95% of samples with VLs loads between 500 and 1000 cp/mL amplified and successfully genotyped (*n* ≥ 10). This criterion had to be modified to sensitivity for amplification ≥95% of samples with VLs > 2000 cp/mL must be amplified and successfully genotyped (*n* ≥ 10) as ViroSeq (our “gold-standard”) is not suitable for VLs < 2000 cp/mL.

Performance metrics used to assess the new method were as follows: (1) accuracy; (2) precision/reproducibility; (3) sensitivity.

### 2.8 Accuracy

Using sensitivity or specificity calculations in the context of sequencing comparisons are impractical and misleading as there are no “real” true negatives especially when including minor mutations in an RNA virus with a low barrier for genomic change.

Therefore, the Jaccard index (J) was employed, which gauges the diversity and similarity of samples. The Jaccard index measures similarity between finite sample sets and is defined as the size of the intersection divided by the size of the union of the sample sets.

J(ViroSeq,veSEQ-HIV)=(|ViroSeq∩veSEQ-HIV|)/(|ViroSeq∪veSEQ-HIV|)

By design values are between 0 and 1 with higher values indicating greater similarity.

Level of agreement between the new test and the reference test (pairwise comparison) was assessed by comparing consensus nucleotide sequences, amino acid sequences, subtype, major, and minor mutations detected. To compare nucleotide sequences, both veSEQ-HIV data and ViroSeq FASTA files were mapped to reference HBX2-HIV using clustalW.^21^ Relevant regions of the *Pol* region (1.3kbp) were extracted from the aligned files. Bases common to both datasets including “ambiguous” bases (IUPAC ambiguity code) in ViroSeq data, were extracted as well as mutations through pairwise comparisons.

As per the manufacturer’s instructions, a cutoff of 30% was used to call an “ambiguous” base for ViroSeq.

### 2.9 Linearity, limit of detection and limit of quantification

Both the limit of detection (LoD) and limit of quantification (LoQ) were assessed using the commercial reference dilution series comparing estimated VL to the number of reads mapping to the reference (HBX2-HIV). Although, veSEQ-HIV is capable of estimating VL based on mapped reads, provided a panel of quantification standards is included in each run, this was not formally assessed given the predicted number of samples in our setting.^10^

Pearson’s correlation co-efficient was used to assess the relationship between VL (cp/mL) and a number of mapped reads.

### 2.10 Precision/reproducibility

Inter- and intra-run agreement was assessed by running replicates on different runs and within the same run. For intra-run comparison, a set of five samples were included on the same run across three different runs; VLs ranged between 3475 and 100 000 cp/mL and included subtypes B, C, and CRF01_AE. For inter-run comparison, a set of eight samples were included on two different runs; VLs ranged between 3435 and 100 000 cp/mL and included subtypes B and CRF01_AE. A summary of replicates can be found in Table 2 below.

WHO recommended (3 × 5 replicates) to determine inter-run precision but due to limited remaining plasma from previously tested samples, a 2 × 8 replicates approach was used.

### 2.11 Specificity

Specificity was assessed by a negative control (PCR HIV negative plasma) included in all runs. Subsequent routine clinical runs have included a phosphate-buffered saline negative control. Possible interfering substances were not assessed as sequencing would only be performed on PCR-confirmed HIV positive samples.

### 2.12 Quality control criteria

A set of quality control (QC) criteria were established to assess both run and sample data quality before analysis. We included sequencing run metrics specified by the manufacturer (Illumina) and criteria to review individual raw read data, inclusive of the negative control. For a review of individual sample data, we referred to guidelines established by the Winnipeg Consensus for read QC, read alignment, reference mapping, HIVDR interpretation, and reporting.^19^

### 2.13 Clinical reporting

Development of our clinical reporting strategy involved discussion with treating clinicians in our network as well as incorporating accreditation requirements, WHO recommendations, and guidelines established by the Winnipeg Consensus.^19,20^ Definitions of major and minor DRMs are as per Stanford University HIVdb.^3–5^

## 3 Results

### 3.1 Accuracy

#### 3.1.1 Accuracy of nucleotide sequences

Pairwise analysis of the *Pol* region resulted in an overall Jaccard index of 0.998, with 98 459 bases shared and 145 single nucleotide polymorphisms (SNPs) difference between sample pairs.

#### 3.1.2 Accuracy of subtyping

Concordant subtypes were achieved for 98% (*n* = 65/66) of patient samples. One sample was misidentified as subtype CRF_14_BG rather than subtype B. This sample had low VL (2790 cp/mL), resulting in less than 1000 mapped reads and suboptimal coverage across the PR gene. Provided samples obtained >1000 mapped reads, 100% accuracy of subtyping could be obtained.

#### 3.1.3 Accuracy of major drug mutations

Due to the low prevalence of major drug mutations and success of current ART, a limited repertoire of major drug mutations could be sourced and included 5 different PR, 19 RT, and 5 IN major mutations. There were 19 (*n* = 19/95) discordant major drug mutation calls between the two methods, however, all discordances were able to be resolved. Most discordances (*n* = 9) were related to the ViroSeq electropherogram where an “ambiguous” base was assigned with reporting calling both wild-type and a DRM. These were all present on veSEQ-HIV data but at a sub-consensus level (defined when <50% but >5% of reads mapping to the alternative allele at a >20-fold read depth). The remaining discordance were as a result of: (1) incorrect manual base assignments on ViroSeq (*n* = 5) and (2) no coverage across region of interest on veSEQ-HIV (*n* = 5). No false positive DRMs were detected on veSEQ-HIV. Taking these additional results into account, the overall accuracy/concordance for detection of major mutations was 100% (*n* = 95/95) with a Jaccard index of 1.0. A summary of these results can be found in Table 3.

#### 3.1.4 Accuracy of minor drug mutations

Only accuracy for PR and RT were examined. Three (3/601; 0.4%) and 29 (29/1249; 2%) discordant minor PR and RT mutation calls were identified, respectively, while 1 (1/569; 0.2%) and 13 (13/2499; 1%) potential veSEQ-HIV false positives were identified. Most false positives (*n* = 10) minor mutations occurred on the boundaries of ViroSeq amplicons resulting in higher uncertainty of base calls.

Irrespective, the overall accuracy/concordance for the detection of minor mutations was 98%. The overall Jaccard index was 0.975. A summary of the concordance of minor drug mutations is available in Supporting Information: Table S4.

### 3.2 Limit of detection and limit of quantification

#### 3.2.1 By viral load

**Figure 1:**
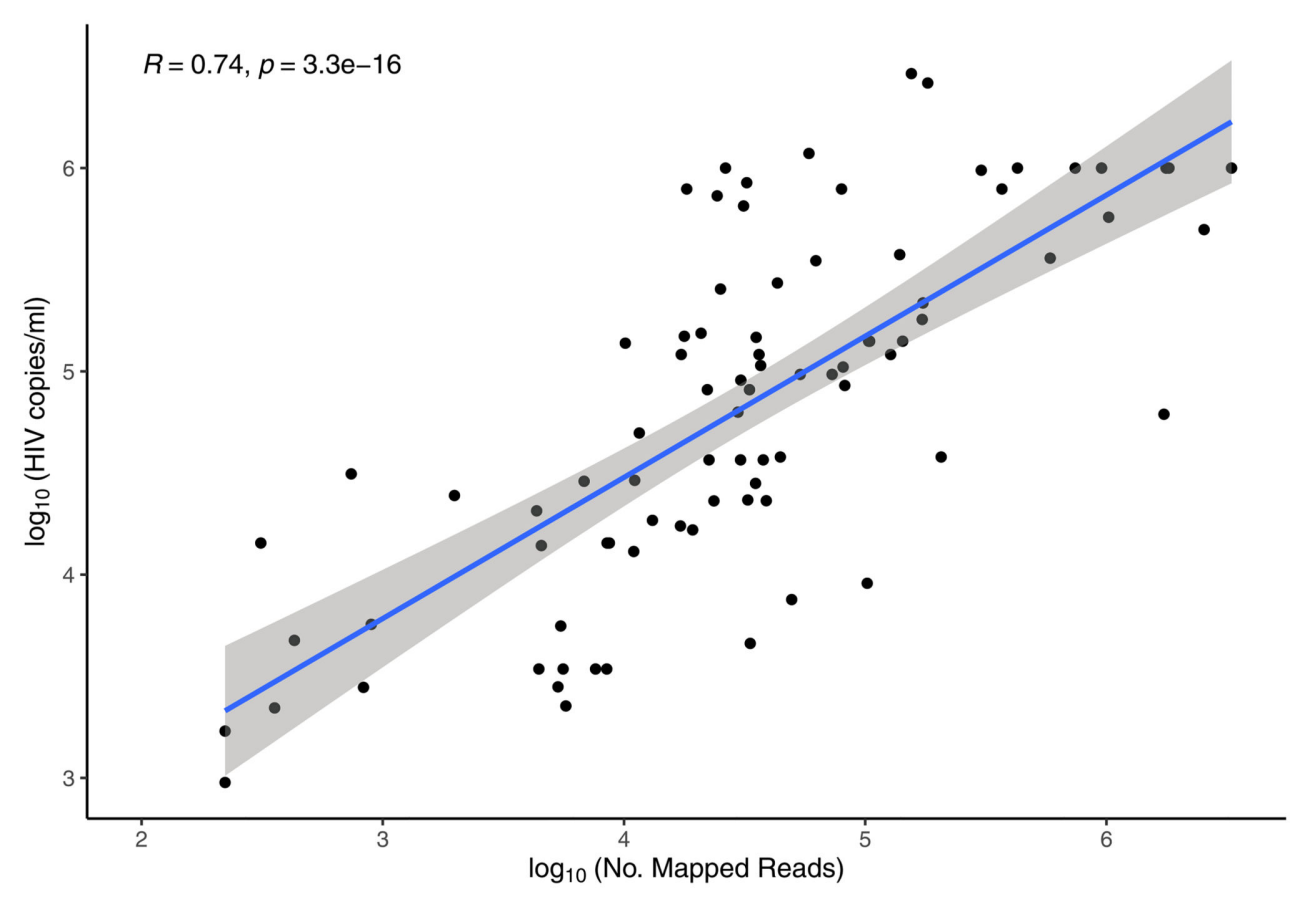
Correlation between viral load (cp/mL) and the number of mapped reads in the subset that passed QC (*n* = 78).

A significant linear correlation (*R* = 0.74; *p* < 0.01) between VL and number of mapped reads (Figure 1) was detected. Lower VLs (2000–5000 cp/mL) gave inconsistent results but were linked to the number of mapped reads with <1000 mapped reads in six of 13 isolates (46%). The remaining seven represented possible failed veSEQ-HIV sequencing, that is, <1000 mapped reads despite VL > 1000 cp/mL. No further sample was available for repeat testing or re-quantification so it was not possible to exclude RNA degradation as the cause. All samples had been stored with an unknown number of freeze-thaw cycles.

#### 3.2.2 By mapped reads

Eight samples (8/82; 10%) run on veSEQ-HIV had suboptimal coverage (<1000 mapped reads). Five (5/8; 63%) of these samples had low VLs (2000–5000 cp/mL). The remaining three had VLs ranging from 5695 to 31 300 cp/mL. As outlined above, we were unable exclude RNA degradation as the cause of suboptimal coverage. The depth of coverage affected all regions when <1000 mapped reads were obtained while > 1000 mapped reads gave excellent coverage depth for all regions. Results are summarized in Table 4.

#### 3.2.3 Linearity

There was a significant linear relationship (*R* = 0.99; *p* < 0.001) between estimated VL and mapped reads. As VL decreased there was a reduction in: (1) the percentage coverage across genes and (2) the number of mutations detected. At VLs <2000 cp/mL potential false-positive mutations were detected (Supporting Information: Table S1). The LoD for HIV-1 was determined to be 100 cp/mL, however, this did not provide adequate mapped reads for reporting. With respect to reporting, the number of mapped reads was the best correlate, requiring a minimum of 1000 mapped reads.

#### 3.2.4 Inter-run (reproducibility) and intrarun (repeatability) of veSEQ-HIV method

Inter-run variability was within the expected performance across the whole genome (*J* = 0.993) and the *Pol* gene (*J* = 0.999) with an average between runs of 437 SNPs and 4 SNPs, respectively.

Similarly, intra-run variability was adequate across the whole genome (*J* = 0.972) and across the *Pol* gene (*J* = 0.999) with an average between runs of 130 SNPs and 4 SNPs, respectively. These results are summarized in Table 2.

#### 3.2.5 Specificity

No evidence of contamination across validation runs or subsequent clinical runs was detected with an average of 20 (range 0–548) reads for the negative control. Although reads represented HIV in a small subset, all negative control assemblies failed and mapping to the reference (HBX2-HIV) was unsuccessful, with 0% coverage and 0% depth across the three regions (PR, RT, and IN) of the genome.

#### 3.2.6 Quality control criteria

QC metrics and their corresponding limits/criteria for sequencing runs are presented in Supporting Information: Table S2.

#### 3.2.7 Clinical reporting

In the transition to NGS-based methods, WHO recommends presenting resistance results in the same way as SS-based methods.^20^ Thus, reports include the same data output generated by ViroSeq-subtype identification and listing identified DRMs and their associated susceptibility interpretations.

Despite the clinical relevance of sub-consensus variants of <20% frequency still being under debate, interest was expressed from treating clinicians in our network to report their presence. Thus, when present with a frequency greater than 5% and with appropriate caveats, sub-consensus variants containing major DRMs are also reported.^19^ These findings are presented in an iterative way with additional mutation scores and interpretation changes reported alongside consensus interpretations. This leaves the choice to consider changes in interpretation to the treating clinician. An example report can be found in Supporting Information: File S1.

## 4 Discussion

In this study, we provide our “in-house” validation of veSEQ-HIV for HIVDR testing in an Australian clinical microbiology laboratory. veSEQ-HIV with minor modifications, met our pre-specified validation criteria.^20^ We, therefore, deemed the assay fit for purpose and able to replace ViroSeq in our laboratory.

We achieved a Jaccard index (*J*) of 0.998 for pairwise nucleotide comparisons of the *Pol* gene and detected 100% of major mutations (Criteria 1: Accuracy 100% of known mutations detected).

A limitation of our validation includes the small number of mutations encountered. We only encountered (11/56; 19%) NRTI; (8/49; 16%) NNRTI, 5/57; 9% PR, and 3/43; 7% IN DRMs (all DRMs encountered are listed in Supporting Information: Table S3). Ideally, our validation set would have included all known mutations that affect clinical interpretation, that is, all known DRMs, however, practically, this is not feasible given the number of known mutations.^20^ Ongoing clinical testing should add to the DRMs encountered.

ViroSeq is not validated for low-level HIV-1 viraemic samples (VL < 2000 cp/mL), and thus the performance of veSEQ-HIV at these levels could not be formally assessed. Nevertheless, for 5 of 6 (83%) subsequent clinical samples with VLs between 1000 and 2000 cp/mL, veSEQ-HIV generated adequate data for resistance determination (data not shown).

Pairwise comparison exhibited low inter-and intra-run variability across the whole genome (*J* = 0.993; *J* = 0.972, respectively). Agreement was highest when limited only to the *Pol* gene (both *J* = 0.999) (Criteria 2: ≥90% of pairwise comparisons must be ≥98% identical). This supports previous findings of relative conservation across the *Pol* gene relative to the rest of the genome.^22^

100% of subtypes were correctly identified (Criteria 3: Sensitivity for amplification: ≥95% of samples with VLs >2000 cp/mL successfully amplified and genotyped (*n* ≥ 10)). There were four subtypes (B, C, CRF01_AE, and CRF02_AG) represented in our sample set. Most samples were subtype B (*n* = 48/66; 72%) followed by subtype CRF01_AE (*n* = 20/66; 18%). This is in keeping with previous Australian epidemiological data identifying subtype B as the most prevalent and CRF01_AE as one of the dominant non-B subtypes of increasing prevalence.^23^ This indicates that while we could not equally represent all HIV genotypes due to lack of sample availability, the subtypes in our sample are representative of circulating HIV in Australia. Irrespective, we do not expect there to be negative bias in “uncommon” genotypes as veSEQ-HIV probes have been designed and previously shown to consistently recover genomes from these “uncommon” HIV-1 subtypes, including C, A (A1 &A2), D, G, and J.^10^

Utilizing the whole genome to determine subtypes provides greater accuracy, especially for recombinant subtypes.^24^ However, discrepancies in subtype determination have been shown to arise based on the region and proportion of the genome used as well as with a choice of subtyping tool.^24^ Therefore, despite having the whole genome available to us, we did not use it to determine subtype as part of this validation.

The arrival of new HIV-capsid antiviral, lenacapavir, highlights the utility of veSEQ-HIV to produce whole-genome sequences in that we have the potential to include this analysis in future clinical reports. Formal assessment, however, will require a different approach as no “gold standard” is available for comparison.

veSEQ-HIV is now in routine use in our laboratory and has been used to successfully sequence and generate over 200 HIVDR and genotyping reports for clinicians across the state of New South Wales, Australia.

Running limited batches (eight patient samples) has allowed easy implementation of the assay into the workflow of our small sequencing laboratory with minimal staff and equipment and has been an ideal use of a small desktop sequencer such as the Illumina iSeq. 100. Despite small batches, reagent cost per assay is still significantly reduced compared to the previous commercial assay (from $360 to $200). Assay batching flexibility also allows for future throughput increases by utilizing automated liquid handling for library preparation and larger sequencing platforms (e.g., Illumina MiSeq).

Processing NGS data has been identified as a major hurdle in the implementation of NGS-based HIVDR. The Winnipeg Consensus has established preliminary guidelines for read QC, read alignment and reference mapping, variant calling and QC, HIVDR interpretation and reporting, as well as general analysis of data management.^19^ These guidelines were invaluable in developing our bioinformatics, QC, and clinical reporting strategy.

Further hurdles for NGS-based HIVDR include a lack of specifically developed external quality assessment (EQA) strategies and programs^8,19^ as current HIVDR EQA are still designed for SS-based methods. In the transition to NGS-based methods, WHO has recommended configuring NGS methods to present results in the same way as SS-based methods.^20^ Thus, while not ideal, NGS-based HIVDR can still be used to produce results formatted as SS-based HIVDR outputs, permitting participation in current EQA programs such as the HIVDR programs available from Quality Control for Molecular Diagnostics (QCMD).

Informal sample exchange between laboratories offers an alternative transitional EQA strategy to meet accreditation needs, however, very few laboratories are currently performing NGS-based HIVDR in Australia. We look forward to more laboratories taking up NGS-based methods to make this another transitional EQA option.

Despite this, there remains an urgent need for appropriate EQA programs and materials to be made available as these approaches overlook the abundance and available complexity in NGS HIVDR data, particularly its ability to detect sub-consensus variants at low levels.^8^

In addition, appropriate reference materials for validation of subconsensus variant detection do not yet exist. Our reporting strategy for sub-consensus variants followed recommendations from the Winnipeg consensus with a 5% frequency threshold suggested to account for errors/bias introduced during assay steps such as RT-PCR, PCR, sequencing steps, etc.^19,25^ While we did not quantify the sensitivity of variant detection in this validation, this threshold is likely conservative with estimates of minority variant frequency likely to be more robust using the probe-based veSEQ-HIV method as biases introduced by PCR are minimized and contamination is computationally controlled.^10^ Our reporting includes necessary caveats to sub-consensus variant reporting and leaves choice to act on subsequent drug susceptibility interpretation changes to the treating clinician.

Well-characterized sample panels, including both clinical and synthetically constructed samples containing sub-consensus variants, are needed to assess each laboratory’s ability to detect sub-consensus variants accurately.^8^

## 5 Conclusion

veSEQ-HIV assay provides a robust, “future-proof” and cost-effective NGS method for HIVDR in a diagnostic setting. veSEQ-HIV demonstrated satisfactory and consistent performance, including low inter-and intrarun variability and good accuracy in detection of DRMs in samples with >1000 mapped reads (achievable in samples with VL > 1000 cp/mL) when compared to “gold-standard” SS-based ViroSeq. veSEQ-HIV met all our pre-set criteria based on WHO “Recommended methods for validating an in-house genotyping assay for surveillance of HIV drug resistance” and has successfully replaced ViroSeq in our laboratory.

The assay meets current needs for clinical reporting (as SS-like results) to meet the requirements of our diagnostic laboratory accreditation and to participate in current EQA programs as well as the ability to report on the presence of sub-consensus variants with appropriate caveats. While currently only the *Pol* region is utilized in our analysis and reporting, veSEQ-HIV recovers whole HIV genomes. As HIVDR transitions from SS-based methods to NGS, new ART drugs with targets other than *Pol* become available, and the clinical relevance of sub-consensus variants is further clarified, veSEQ-HIV meets both current and future needs for HIVDR testing in a diagnostic setting.

## Supplementary Material

Example Clinical Report

Probe Sequences

Supplementary Data

## Figures and Tables

**Table 1:** Sample baseline characteristics.

Subtype	Number of samples	Mean viral load (cp/mL)	Viral load range	Number surveillance drug resistance mutations		
				Protease (PR)^a^	Reverse-transcriptase (RT)^a^	Integrase (IN)^b^
B	48	364 275	2260–2 914 185	6	54	5
C	3	41 444	14 309–81 222	0	5	0
CRF01_AE	13	167 506	1700–847 000	0	26	0
CRF02_AG	2	53 025	1050–105 000	0	4	0

a Surveillance drug resistance mutations as determined by ViroSeq assay.

b Only 5/66 (8%) samples had integrase testing at baseline. The integrase (IN) region is not covered by the ViroSeq assay. When requested IN testing was referred to an external laboratory who performs an SS-based in-house assay and utilize the Stanford HIVdb Program for analysis.

**Table 2:** Intra- and inter-run comparison including Jaccard index and SNP numbers across whole-genome and across *Pol* gene.

	Number of samples	Replicates per sample	Viral load (cp/mL)	Subtypes	Jaccard index-whole genome (mean SNPs)	Jaccard Index-Pol gene (mean SNPs)
Intra-run	5	3	3435–100 000	B, C, CRF01_AE	0.972 (130)	0.999 (4)
Inter-run	8	2	3435–100 000	B, CRF01_AE	0.993 (437)	0.999 (4)

Abbreviation: SNPs, single nucleotide polymorphisms.

**Table 3:** Concordance of major drug mutations between methods.^a^

Major mutation location	Discordant mutation calls	Presence of sub-population	ViroSeq poor coverage/false calls	veSEQ-HIV poor coverage	Discordance resolved (%)	Overall accuracy (%)
Protease (PR)	3	1	2	-	100 (*n* = 3/3)	100 (*n* = 6/6)
Reverse-transcriptase (RT)	16	8	3	5	100 (*n* = 16/16)	100 (*n* = 84/84)
Integrase (IN)	-	-	-	-	-	100 (*n* = 5/5)^b^
Total	-	9	5	5	100 (*n* = 19/19)	100 (*n* = 95/95)

a Replicates were included in mutation analysis.

b Sanger sequencing integrase results only available for 5/66 samples.

**Table 4:** veSEQ-HIV QC metrics based on number of mapped reads.

No. mapped reads	No. of samples	Average depth coverage		
		Protease (PR) region	Reverse-transcriptase (RT) region	Integrase (IN) region
<1000	8	71% @ 9 fold	42% @ 8 fold	52% @ 7 fold
>1000	78	99% @ 2914 fold	99% @ 3465 fold	99% @ 3372 fold

## Data Availability

The data that support the findings of this study are available from the corresponding author upon reasonable request.
