# Supplementary material for: Validation of an HIV whole genome sequencing method for HIV drug resistance testing in an Australian clinical microbiology laboratory
Source: J Med Virol. Author manuscript; Available in PMC 2026 Aug 10. (PMC7619347; doi:10.1002/jmv.29273)
Supplement: Supplementary file 1 [file EMS217196-supplement-Example_Clinical_Report.pdf]

Supplementary file 1: Example Clinical Report

Royal Prince Alfred Hospital

Missenden Road, Camperdown, NSW 2050

Department of Microbiology:

Enquires:

HIV-1 Sequencing Report

Name of Patient:

MRN: **Date of Birth:**

Date of Collection: **Referring Doctor:**

Provided HIV Viral Load :

Our Reference / Accession: **Date of Analysis:**

Referring Laboratory: **Referring Lab. Reference:**

Sequencing Contact Staff:

Clinical Enquiries:

Methodology:

Capture based whole genome sequencing was performed on this sample. HIV-1 RNA was extracted followed by a one-step RT-RNA PCR prior to sequencing using Illumina technology. Consensus genome was generated using Shiver v 3 specifying a minimum depth of 5-15 against the reference genome (Human immunodeficiency virus type 1 - HBX2)[1]. These thresholds have been validated against previous Viroseq results and recommended bioinformatic strategies[2]. Drug resistance mutation(s) were subsequently identified using the web-interface tool sierrapy against the Stanford University db [version(s): Sierra 3.4.4 (2023-06-11)]. Variant frequency for major drug-resistance mutations are determined with sub-consensus variants (at a frequency > 5%) indicated in the table. Potential drug susceptibility changes are based on Stanford major resistance mutations.

The effect(s) on drug resistance with the inclusion of sub-consensus mutations are shown in the table(s) under "score +" and "interpretation change" column(s). Minor variants are reported only if represented in the majority-population.

1. Wymant C. et al. Virus Evolution, Volume 4, Issue 1, January 2018, vey007. https://doi.org/10.1093/ve/vey007
2. Ji H. et al. Journal of the International AIDS Soceity, 2018, 21:e25193. https://doi.org/10.1002/jia2.25193

Results:

No. reads mapped: 841138

HIV-1 subtype (Stanford db) : B (3.38%)

QC metrics across HIV-1 genes

Gene	Coverage (%)	Av. Depth (No. reads)
**PR**	98.99	16556
**RT**	99.82	14471
**IN**	99.65	25440

The confidence of excluding HIV-1 gene resistance mutations (i.e. determining drug-class susceptibility) increases with overall gene coverage (>50%) and read depth (>15). Subsequent interpretations are based on aligned and sequenced data. Repeat testing in the setting of low viral loads (i.e. mapped reads) is unlikely to provide greater confidence.

Drug-resistance mutations:

Mutation	Gene	Stanford Type	Total No Reads	Ref Coverage	Mutation Coverage (%)
**K101P/Q/T**	RT	NNRTI	5153	3907 (75%)	1316 (25%)*
**E138K**	RT	NNRTI	7174	4309 (60%)	2910 (40%)*

Coverage corresponds to the number of reads supporting the reference and variant or deletion.
A few studies have been able to demonstrate sub-consensus variants (<20% frequency) contribute to negative clinical outcomes, however, the overall clinical relevance of detection of these variants is unknown.

Drug-susceptibility interpretations - Protease (PR)

class	name	score	interpretation
PI	atazanavir (ATV/r)	0	Susceptible
PI	darunavir (DRV/r)	0	Susceptible
PI	fosamprenavir (FPV/r)	0	Susceptible
PI	indinavir (IDV/r)	0	Susceptible
PI	lopinavir (LPV/r)	0	Susceptible
PI	nelfinavir (NFV)	0	Susceptible
PI	saquinavir (SQV/r)	0	Susceptible
PI	tipranavir (TPV/r)	0	Susceptible

Major / Accessory (PR) Mutations: None detected
Other Mutations: L10V; T12P; K14R; I15V; M36I; N37D; L63P; I93L

Please note: Drug-susceptibility interpretations in this example report only include the Protease region. Reverse Transcriptase and Integrase regions are reported in a similar manner.
