# Supplementary material for: Validation of an HIV whole genome sequencing method for HIV drug resistance testing in an Australian clinical microbiology laboratory
Source: J Med Virol. Author manuscript; Available in PMC 2026 Aug 10. (PMC7619347; doi:10.1002/jmv.29273)
Supplement: Supplementary file 2 [file EMS217196-supplement-Supplementary_Data.docx]

**Supplementary File 3**

**Supplementary Table 1. veSEQ-HIV results of dilution series of commercial standard AcroMetrix HIV-1 Panel**

| **Estimated viral load (cp/mL)** | **Mapped reads** | **Subtype** | **Protease %** | **Reverse-Transcriptase %** | **Integrase %** | **No. Mutations detected** |
| --- | --- | --- | --- | --- | --- | --- |
| 1000000 | 937160 | B | 99 | 100 | 100 | 22 |
| 100000 | 47356 | B | 99 | 100 | 100 | 22 |
| 10000 | 8766 | B | 99 | 100 | 100 | 22 |
| 1000 | 1086 | B | 99 | 83 | 65 | 19 & 1 FP |
| 100 | 114 | B | 46 | 18 | 32 | 1FP |
| 50 | 10 | B | 12 | 0 | 0 | 0 |
| 25 | 20 | B | 75 | 0 | 0 | 0 |
| 0 | 6 | CRF01_AE | 0 | 0 | 0 | 0 |

Supplementary Table 1 depicts the quantification and limit of detection against the commercial standard and sequenced using veSEQ-HIV. The Standard HIV sample (subtype B with drug susceptible profile) contained 20 minor/accessory mutations [Protease = 3; Reverse-Transcriptase = 13 and Integrase = 4].

**Supplementary Table 2. Selected QC metrics for sequencing run/sample analysis**

| **QC metric** |  |  |
| --- | --- | --- |
| **Illumina sequencing run (iSeq100 i1 v2 reagent; 300 cycles;2 x 150bp)** | Occupancy (%)^a^ | ≥80^b^ |
|  | Clusters passing filter (%PF)^a^ | ≥53^b^ |
|  | Overall Qscore (%) | ≥80%^c^ |
|  | PhiX error rate (%) | ≤1 |
| **Negative control** | Mapped reads (HIV-1-HXB-2) | <1000 |
| **Sample quality** | Mapped reads (HIV-1-HXB-2) | >1000 |
|  | Average mapped read quality | >Q30 |
| **HIV-1 genes (Protease, Reverse-Transcriptase, Integrase)^d^** | Gene coverage (%) | >50 |
|  | Read depth | >15 |

^a^ On the Illumina iSeq100, %PF and % Occupancy represent an expected level to maximize data output and data quality. Metrics may fall outside of these ranges but still produce acceptable data as quality can still be high even with a lower quantity of reads.

^b^ QC metric determined by quantification of mean – 3SD for runs included in the validation

^c^ Manufacturer sequencing specifications

^d^ The confidence of excluding HIV-1 gene resistance mutations (i.e. determining drug-class susceptibility) increases with overall gene coverage (>50%) and read depth (>15). **Supplementary Table 3. Major and minor mutations encountered in our sample set**

| **Region** | **Major mutations** | **Minor mutations** |
| --- | --- | --- |
| **Protease** | M46L; G48A; I54V; V82A; L90M | K14R; T12S; N37I; L63P; I62V; T12P; I13V; R41K; E35D; I15V; L10I; T12R; V77I; G16A; P39S; G17E; T12A; T12K; G16E; T4S; T4P; L10V; L19I; T12N; N37S; R41K; I15V; I62V; Q92K; L63P; K45R; N37E; N37S; I13V; L19I; V11A; K20R; E35D; L10I; T12K; M36I; L19Q; L33V; L19T; G16E; L33I; T12A; R57K; K14R; I93L; V77I; Q18H; D60E; G17E; L63Q; L63T; G16A; L63H; Q61E; H69K; L19V; N37A; I64V; N37D; N37T; L63A; I72V; K20M; H69C; I64L; T74A; L63S; N37V; K20I; A71T; E65D; A71V; K43R; M36T; C67S; L89M; I72T; H69Y; N37K; M36V; T74S; H69Q; K70E; L63V; R41N; K70R; Q58E; V82I; L89I; I85V; T91A; I72M; T91V; F99L; I93M |
| **Reverse-Transcriptase** | M41L; K65R; D67N; T69D; K70E; K70T; V75M; V75I; L100I; K103N; E138A; E138Q; Y181C; M184V; G190A; T215D; K219Q; P225H; N348I | K20R; K32E; D123E; V35I; S68G; E6D; A158S; V35T; I50V; V8I; R83K; E6A; V60I; V35L; K22R; I142L; V245R; K122E; K102R; K11R; S48E; P4Q; I135T; A98S; K11Q; D86E; V35T; K173Q; I135V; K103R; K122Q; S162A; T39L; V60I; K64R; K11I; D121H; S68G; K101Q; T39K; Q207E; A98S; K166R; K11T; K49R; D177E; I135T; T200A; S48T; I293V; K104R; R83K; K20R; E40D; T69N; T39S; E6D; D123E; T39R; F171Y; T69A; V35I; V8I; E399D; E291D; L533M; K390R; E404D; S468P; L469I; K366R; G335D; E432D; V435E; K281R; A554S; V435I; Q480H; F346Y; K558R; E514Q; H483Y; K512R; I329V; M357K; D324E; T477A; H483N; N447S; Q334L; T386I; V292I; S519N; R461K; I326V; L491S; R356K; K431S; K530R; L452I; R358K; L469F; A400T; E514D; L503M; A554N; L491P; L517T; A376S; T403M; G359S; V531I; L491A; Q524E; E546D; A554T; A371V; D471E; V559I; H483Q; A534S; T377M; K431T; Q464K; A376T; T470A; E529D; G196E; K277R; V245I; S162C; V189I; I135L; Q174K; K122E; P170T; E248N; V245Q; Q207D; F214L; R172K; D123S; E312A; Q278E; A288S; R211K; I178L; V245E; I178M; M357T; A272P; R211Q; T200I; K43E; I202V; S134G; E169D; I142T; E298A; I142V; Q174N; T286A; K201R; Q334H; Q207A; D121Y; Q207H; T200V; D460N; D121C; L283I; V245M; V466I; T286P; I329L; V179I; K311R; K102Q; K275R; S379C; D250E; E396D; G333E; G436E; V317A; P294S; L228Y; Q334R; E344K; Q334E; T240K; R307K; S322T; F440Y; R211S; K238R; A360T; K350R; I393V; H315Y; A272G; T39A; P294T; E297K; F346C; E297A; E344D; E203D; T200E; L301I; K476Q; R284K; S379G; E297V; K173S; V381I; E312T; Q334S; V435A; H483S; A502V; T377L; Q520K; V467I; E298D; T377R; T403V; E449D; V458I; T470N; R358G; N418S; L517I; I506L; H483L; G490E; R463K; A554K; E516D; V548I; L452Q; I434M; V518I; L517V; I556V; K527N; S468T; Q524K; E492K; K550R; S489A; H483C; S515L; K527Q; L310I; A502G; E492A; R448K; K512Q; K451R; G359T; V435M; S468C; T403R; I434L; K527R; T377V; L452V; K476R; T450S; T386V; K395R; E370D; A400V; I375V; E370A; S251I; L228R; D123N; K173T; I178V; V245N; K173I; I135R; S162Y; I135K; K249R; K385R; V245K; P294Q; V245T; I257L; K173A; K173R; V365I; Q207K; P345Q; D324G; T369A; L325I; S468N; D320E; V276I; P345T; A376V; S322P; Q207S; T296S; L301M; T377Q; T200L; I411V; A400S; K512T; V245A; Q334P; L484I; T470Q; V435T; V417I; T377N; E312N; T369V; Y342F; V435L; E36A; G196R; T39E; K122P; E204Q; K43A; V118I; I94L; K11A; S251T; K173E; I132L; G196V; K102R; D177G; E53D; T39M; E204D; I382V; Q509K; E312S; A508G; L452A; Q394R; E297R; Q174T; G335S; T286V; E248D; E370V; E328D; K465R; F389L; Q524H; Q197H; T386A; A304E; S251C; Q394L; A272S; T470K; A534T; M357R; Q547K; T403I; G335E |
| **Integrase^a^** | N51Y; T97A; Y143C; N155H; N155K | Not examined |

^a^ Sanger sequencing integrase results only available for n=5 samples
**Supplementary Table 4. Concordance of Minor Drug Mutations**

| **Minor mutation location** | **ViroSeq**  **Total mutation calls** | **ViroSeq**  **Discordant mutation calls** | **veSEQ-HIV**  **Total mutation calls** | **veSEQ-HIV**  **Discordant mutation calls** |
| --- | --- | --- | --- | --- |
| **Protease** | 601 | 3 (0.4%) | 569 | 1 (0.2%) |
| **Reverse-Transcriptase** | 1249 | 29 (2%) | 2499^a^ | 13 (1%) |

^a^ 1317 of these mutations are post amino-acid position 320 in the Reverse-Transcriptase gene; a region not amplified in the ViroSeq assay
